# Identification of epithelial-mesenchymal transition prognostic signature associated with prognosis, tumor microenvironment, and therapeutic effect in prostate cancer

**DOI:** 10.3389/fgene.2025.1539745

**Published:** 2025-08-11

**Authors:** Yiyuan Li, Ke Li, Hua Wang, Jianguang Qiu, Chutian Xiao

**Affiliations:** ^1^ Department of Urology, The Sixth Affiliated Hospital, Sun Yat-Sen University, Guangzhou, Guangdong, China; ^2^ Biomedical Innovation Center, The Sixth Affiliated Hospital, Sun Yat-sen University, Guangzhou, Guangdong, China; ^3^ Department of Urology, The Third Affiliated Hospital, Sun Yat-Sen University, Guangzhou, Guangdong, China

**Keywords:** epithelial-mesenchymal transition, prostate cancer, prognostic signature, tumor microenvironment, therapy response

## Abstract

**Background:**

Prostate cancer (PCa) is a prevalent malignancy and a leading cause of cancer-related death among men. Epithelial-mesenchymal transition (EMT) plays a crucial role in tumor progression, metastasis, and treatment. However, there are limited comprehensive studies on the EMT correlation with prognosis, tumor microenvironment, and therapeutic efficacy in PCa.

**Methods:**

We obtained mRNA expression profiles and clinical data of PCa samples, along with 1,011 protein-coding EMT-related genes from public databases. Functional annotation and consensus clustering were performed based on differentially expressed genes. An EMT prognostic signature (EPS) was constructed in the TCGA dataset after a series of bioinformatics analyses and validated in the GSE116918 dataset. The signature was used to explore clinicopathological features, genomic heterogeneity, the immune landscape, and therapy responses. Finally, we examined the expression of key genes in clinical specimens.

**Results:**

An EPS was established based on four key genes (MEN1, H2AFZ, UCKL1, and FUS). The patients were classified into low-risk and high-risk groups according to their median EPS risk scores. In both datasets, patients in the high-risk group exhibited significantly lower survival rates compared to those in the low-risk group. Furthermore, the EPS risk score proved to be an independent prognostic factor, and the prognostic nomogram based on the EPS risk score and T stage yielded high accuracy. Subsequent investigations found that the EPS risk score was correlated with both tumor mutation burden and genomic heterogeneity. Notably, the low-risk group displayed a higher proportion of tumor-infiltrating immune cells and exhibited better responses to chemotherapy and immunotherapy. As expected, the validation analysis confirmed substantial overexpression of MEN1, H2AFZ, UCKL1, and FUS in PCa tissues relative to adjacent normal prostate tissues.

**Conclusion:**

Our preliminary EPS represents a promising biomarker for predicting PCa prognosis and has great potential for clinical application.

## 1 Introduction

Prostate cancer (PCa) is among the most prevalent malignancies in men and poses a major health threat (Bergengren et al., 2023). Globally, approximately 1.41 million new cases and 380,000 deaths are attributed to PCa annually (Sung et al., 2021). In the United States, PCa ranks first in new cases and second in tumor-related deaths among men (Siegel et al., 2024). The prognosis of PCa patients varies greatly depending on metastasis status (Siegel et al., 2020). Most early and localized PCa tends to exhibit a slow growth pattern and generally carries a favorable prognosis. However, patients who developed distant metastases experienced a notable decline in survival rates (Boeve et al., 2019; Kawakami et al., 2021). Consequently, accurate prediction of metastasis becomes pivotal in PCa management. However, the current understanding of metastatic markers for prostate cancer remains inadequate.

The biological process of conversion of epithelial cells into mesenchymal stromal cells, termed epithelial-mesenchymal transition (EMT), greatly contributes to cancer metastasis (Bakir et al., 2020). Tumor cells go through several changes during EMT, such as losing epithelial cell characteristics like cell-cell adhesion and gaining mesenchymal cell traits including higher collagenase and extracellular matrix-degrading enzyme activity (Jonckheere et al., 2022). As a result, tumor cells can breach the basement membrane, invade adjacent tissues, and enter blood vessels (Odero-Marah et al., 2018). Notably, EMT has been associated with metastasis and poor prognosis in several solid malignancies that promote metastasis (Odero-Marah et al., 2018; Liu et al., 2020; Lüönd et al., 2021). In PCa, activation of signaling pathways such as MAPK/ERK and PI3K/Akt can promote invasion and metastasis through EMT (Meng et al., 2021; Li et al., 2022). Furthermore, EMT may contribute to PCa metastasis or recurrence after treatment by enhancing resistance to drugs (Wade and Kyprianou, 2018; Jonckheere et al., 2022). Therefore, investigating EMT in PCa holds promise for predicting metastatic tendencies and unraveling the underlying metastatic mechanisms. Nonetheless, deepening the process of EMT in PCa metastasis poses a significant challenge due to the intricate network operating within the tumor microenvironment (TME) (Odero-Marah et al., 2018) and the dynamic balance that exists between EMT and mesenchymal-epithelial transition (MET) in tumors (Bakir et al., 2020). Therefore, it is worth identifying key genes associated with EMT in PCa.

Here, a comprehensive analysis was conducted to investigate key EMT-related genes associated with PCa. In addition, this study constructed a prognostic signature as an independent factor in PCa patients. Finally, we utilized multiple public databases and functional experiments to validate the stability and reliability of our results. We expect that the present study will provide valuable information for prognostic prediction and personalized medical treatment of patients with PCa.

## 2 Materials and methods

### 2.1 Data sources

The RNA-seq expression profiles, mutation data, and corresponding clinical information of PCa were obtained from The Cancer Genome Atlas (TCGA, https://portal.gdc.cancer.gov/) database, which contained 499 PCa specimens and 52 normal tissue specimens. Characteristics of the PCa patients from the TCGA data were listed in Table 1. Furthermore, from the Gene Expression Omnibus (GEO, https://www.ncbi.nlm.nih.gov/geo/) database, we downloaded gene expression and complete biochemical recurrence (BCR) data for 248 PCa samples in GSE116918 (Jain et al., 2018). Then we validated our results in an independent external validation dataset that combined the transcriptional expression of key prognostic genes and clinical data. We acquired a total of 1,011 protein-coding EMT-related genes from dbEMT 2.0 (https://dbemt.bioinfo-minzhao.org/index.html) (Zhao et al., 2019).

**TABLE 1:** Characteristics of different clusters included in this study.

Variable	Cluster1 (n = 309)	Cluster2 (n = 186)
	Number (%)	Number (%)
Age		
≤60	149 (48.2%)	73 (39.2%)
>60	160 (51.8%)	113 (60.8%)
Fustat		
Dead	3 (1.00%)	7 (3.80%)
Alive	306 (99.00%)	179 (96.20%)
T stage		
T2a	11 (3.56%)	2 (1.08%)
T2b	7 (2.26%)	3 (1.61%)
T2c	132 (42.72%)	32 (17.20%)
T3a	87 (28.16%)	70 (37.63%)
T3b	63 (20.39%)	71 (38.17%)
T4	4 (1.29%)	6 (3.23%)
Unknown	5 (1.62%)	2 (1.08%)
N stage		
N0	218 (70.55%)	126 (67.74%)
N1	31 (10.03%)	47 (25.27%)
Unknown	60 (19.42%)	13 (6.99%)
PSA		
≤10	272 (88.03%)	150 (80.65%)
10–20	7 (2.27%)	4 (2.15%)
>20	1 (0.32%)	4 (2.15%)
Unknown	29 (9.38%)	28 (15.05%)
Gleason Score		
≤6	39 (12.62%)	6 (3.23%)
7	179 (57.93%)	67 (36.02%)
≥8	91 (29.45%)	113 (60.75%)

PSA, prostate-specific antigen.

### 2.2 Differential expression and functional enrichment analysis

Differentially expressed genes related to EMT from TCGA datasets were identified between PCa and normal prostate samples using the ‘limma’ R package. The genes were considered significant if they had an adjusted *P*-value <0.05 and log |fold change (FC)| > 1. The functional enrichment analysis of the differentially expressed EMT-related genes was systematically explored using Gene Oncology (GO) and Kyoto Encyclopedia of Genes and Genomes (KEGG) analyses with the ‘clusterProfiler’ R package (Yu et al., 2012).

### 2.3 Protein–protein interaction network and key modules analysis

The search tool for the retrieval of interacting genes/proteins (STRING) database (http://string-db.org) was used to construct protein-protein interactions (PPI) among the differentially expressed EMT-related genes [18]. The most important module of the PPI network was then identified using the Molecular Complex Detection (MCODE) plugin of Cytoscape with default parameters [19].

### 2.4 Sample clustering based on a non-negative matrix decomposition algorithm

Non-negative matrix factorization (NMF) was used to classify patients into different EMT regulator patterns based on the gene expression profiling of differentially expressed EMT-related genes. The ‘NMF’ R package was utilized for this purpose (Gaujoux and Seoighe, 2010). The number of clusters (k) ranged from 2 to 10, and the optimal number (k = 2) was determined based on factorization ranking parameters such as cophenetic and dispersions as well as consensus heatmap. The study analyzed the differences in progression-free survival (PFS) between distinct clusters. Additionally, the immune infiltration landscape of different clusters was elucidated using the single-sample gene set enrichment analysis (ssGSEA) algorithm (Subramanian et al., 2005). The gene expression and the clinicopathological distribution of the clusters were also visualized using the heatmaps. Finally, the expression level of PD-1 was evaluated across different PCa patterns.

### 2.5 Construction and external validation of the EMT prognostic signature

Differentially expressed genes (DEGs) were identified between the different EMT regulator patterns, with an adjusted *P*-value <0.05 and log |fold change (FC)| > 2. The genes related to the EMT regulator pattern were then analyzed using univariate Cox regression analysis to determine their association with PFS. Additionally, patients were divided into different gene clusters based on the expression of prognostic DEGs using the NMF unsupervised clustering approach.

The EMT prognostic signature (EPS) was constructed using the TCGA dataset. To minimize over-fitting prognostic characteristics, the Least Absolute Shrinkage and Selection Operator (LASSO) regression analysis was employed through the ‘glmnet’ R package. The study identified key prognostic genes through multivariate Cox analysis and constructed an EPS using the formula: risk score = ∑ (Coefi*Expi), where Coefi and Expi represent the regression coefficient and corresponding expression value of each gene. According to the median risk score, 595 patients in the TCGA dataset were sorted into low-risk group and high-risk group, and then subjected to survival analysis. Furthermore, the performance of the constructed EPS was tested using time-dependent receiver operating characteristic (ROC) curve analysis, principal component analysis (PCA), and t-distributed stochastic neighbor embedding (t-SNE) with the ‘timeROC’ and ‘Rtsne’ R packages. The EPS’s utility was validated using the GSE116918 dataset as an independent external cohort. With the 'survminer’ R program, an optimal cut-off value for survival analysis was established.

### 2.6 Correlation analysis of clinicopathological features and development of clinical nomogram

To determine the practical utility for the EPS, correlation analyses, and stratified survival analyses in the TCGA dataset were carried out. Next, to ascertain if the signature may function as an independent prognostic factor, we conducted univariate and multivariate Cox regression analyses. A clinical nomogram was developed using the 'rms’ R package to predict PFS by combining independent prognostic factors. The precision of the nomogram prediction outcomes was evaluated with calibration curves as well as decision curve analysis (DCA) via the 'ggDCA’ R package.

### 2.7 Genomic heterogeneity analysis

The tumor mutation burden (TMB) value of each PCa sample was calculated with the 'maftools’ R package (Mayakonda et al., 2018) after downloading somatic variant data. Additionally, we conducted differential and survival analyses of TMB between two risk groups. We also utilized Pearson’s method to examine the correlation between the risk score and various genomic heterogeneities, including homologous recombination defect (HRD), loss of heterozygosity (LOH), microsatellite instability (MSI), purity, mutant-allele tumor heterogeneity (MATH), neoantigens, and ploidy (Bonneville et al., 2017; Thorsson et al., 2018).

### 2.8 Immune landscape analysis

Immune infiltration scores were assessed by currently accepted methods, such as TIMER (Li et al., 2020), CIBERSORT (Chen et al., 2018), CIBERSORT-ABS (Tamminga et al., 2020), QUANTISEQ (Chen et al., 2018), MCPCOUNT (Dienstmann et al., 2019), XCELL (Aran et al., 2017), and EPIC (Racle et al., 2017). The relative abundance of immune cells between different risk groups of PCa patients was compared by a ssGSEA. Additionally, the relative contents of 22 tumor-infiltrating immune cells (TIICs) were obtained with the CIBERSORT algorithm. We then conducted correlation analyses between the risk scores and the TIIC contents. Finally, we analyzed the relationship between the two risk groups and the immune subtypes reported by Thorsson et al. This study conducted an immunogenomic analysis of more than a thousand tumor samples covering 33 different cancer types and defined six immunological categories (C1-C6) (Thorsson et al., 2018).

### 2.9 Immunotherapy response and chemotherapy sensitivity analysis

We compared the expression levels of 46 immune checkpoints between two risk groups. Then, we utilized the Cancer Immunome Atlas (TCIA, https://tcia.at/home) database (Charoentong et al., 2017) to quantitatively measure tumor immunogenicity using the immunophenoscore (IPS), which ranges from 0–10. To identify the relationship between drug sensitivity and risk score, we used the “oncoPredict” R package to predict the five most common drug sensitivities (Maeser et al., 2021). The difference in sensitivity score was then compared between the two groups with Wilcoxon signed-rank tests. Subsequently, the anti-cancer drug targets were extracted from the DrugBank (https://go.drugbank.com/) database (Wishart et al., 2018) and expression differences were also analyzed between the two groups. The association of the key prognostic gene with chemotherapeutic drug sensitivity was explored to validate the efficacy of these genes in predicting drug sensitivity using the Gene Set Cancer Analysis (GSCA, https://guolab.wchscu.cn/GSCA) database (Liu et al., 2023).

### 2.10 Validation of protein expression and mRNA levels of key prognostic genes

The protein expression levels of the key prognostic genes in PCa relative to normal tissue were measured via immunohistochemistry (IHC) staining in the Human Protein Atlas database (HPA, https://www.proteinatlas.org/) (Asplund et al., 2012). The IHC images were downloaded from this database. The IHC images were analyzed by calculating the percentage of the reaction areas using ImageJ software, version 1.53k, (Wayne Rasband, the National Institutes of Health in the USA). Data visualization was conducted with the GraphPad Prism software, version 9.0.0.

Additionally, tissues from ten PCa patients were obtained for quantitative real-time polymerase chain reaction (qRT-PCR) assays. This study was approved by the Ethics Committee of The Sixth Affiliated Hospital of Sun Yat-sen University (the Ethical Approval Number: 2022ZSLYEC-468). All patients signed written informed consent forms to use their histopathological samples for research purposes, and the study was conducted in accordance with the Declaration of Helsinki. Total RNA was extracted from cells using TRIzol™ Reagent (Invitrogen, USA) according to the manufacturer’s instructions. cDNA was synthesized from 1 μg of total RNA using a reverse transcription kit (please specify the kit and manufacturer, if available). Quantitative real-time PCR (qRT-PCR) was performed using SYBR GreenER™ qPCR SuperMix (Invitrogen, USA) on a Bio-Rad iCycler system (Bio-Rad Laboratories, CA, USA). The thermal cycling conditions were as follows: initial denaturation at 95°C for 1 min, followed by 35 cycles of denaturation at 95°C for 90 s, annealing at 60°C for 30 s, and extension at 72°C for 30 s. A final extension was performed at 72°C for 10 min. The relative mRNA levels were standardized using the 2^−ΔΔCT^ technique and compared to GAPDH. The qRT-PCR primer sequences can be found in Table 2.

**TABLE 2:** Primer sequences for qRT-PCR.

Gene	Primer sequences
MEN1-F1	GTG​GCC​ACC​AAG​ATC​AAC​TC
MEN1-R1	CCG​CTT​GAG​GAA​AGA​CAG​A
H2AZ1-F1	ACT​TGA​ACT​GGC​AGG​AAA​TG
H2AZ1-R1	GCC​TTG​ATG​AGA​GAA​TCC​A
UCKL1-F1	CAG​TCG​CGA​CGA​GTT​CAT​CT
UCKL1-R1	GTG​ATC​TGC​TTC​CCC​GCA​TA
FUS-F1	TCA​CGT​CAT​GAC​TCC​GAA​CA
FUS-R1	CTC​CCT​TCA​GCT​TGC​CAG​TT
GAPDH-F1	GGGAAACTGTGGCGTGAT
GAPDH-R1	GAG​TGG​GTG​TCG​CTG​TTG​A

## 3 Results

### 3.1 Differentially expressed EMT-related genes in prostate cancer and functional enrichments analysis

**FIGURE 1:**
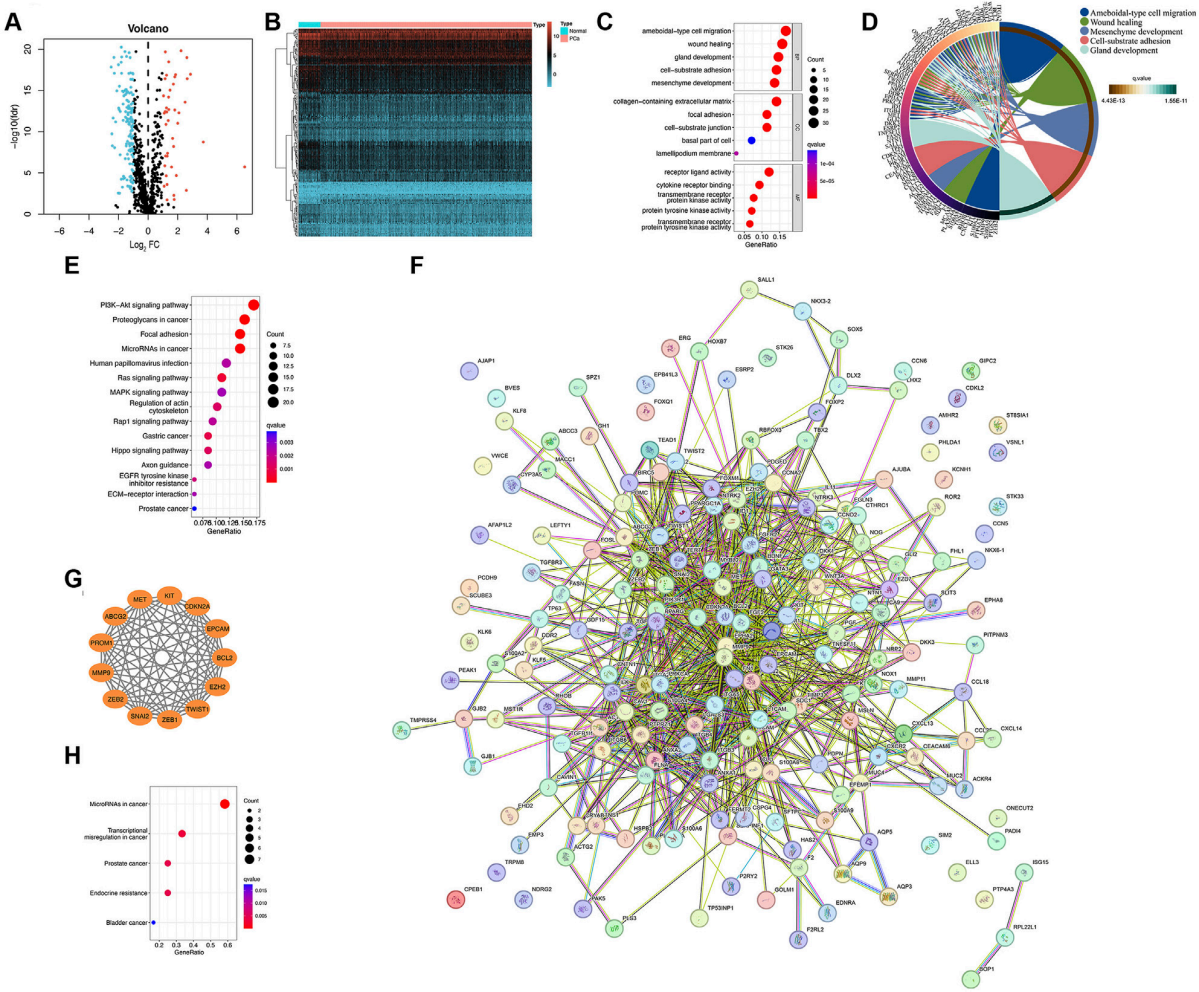
Transcriptional alterations of EMT-related genes and functional enrichment analysis in PCa. **(A,B)** Heat map and volcano map of differentially expressed EMT-related genes in the TCGA cohort, and differential expression analysis identified 186 EMT-related DEGs, with 42 upregulated DEGs and 144 downregulated DEGs in PCa relative to normal tissue. **(C)** GO annotation of 186 differentially expressed EMT-related genes. **(D)** Leading edge analysis of 186 differentially expressed EMT-related genes. **(E)** KEGG annotation of 186 differentially expressed EMT-related genes. **(F)** PPI network constructed with the STRING database. **(G)** The most significant module was obtained from the Cytoscape plugin MCODE. **(H)** KEGG pathway analysis of the genes from the most significant module.

Information on EMT-related genes in *homo sapiens* was obtained from the dbEMT2.0 database, with 1,184 genes identified. Of these, 85.4% (n = 1,011) were retained for subsequent analysis. Transcriptome data for these genes were obtained from the TCGA database. Differential expression analysis identified 186 EMT-related DEGs, with 42 upregulated DEGs and 144 downregulated DEGs in PCa relative to normal tissue (Figures 1A,B).

Functional enrichment analysis was utilized to investigate the molecular mechanisms related to the EMT-related DEGs. The biological processes were associated with gland development, cell-substrate adhesion, and mesenchyme development. The cellular component was found to be involved in the collagen-containing extracellular matrix, focal adhesion, and cell-substrate junction. The molecular function was related to receptor-ligand activity, cytokine receptor binding, and transmembrane receptor protein kinase activity (Figure 1C). Leading edge analysis of 186 differentially expressed EMT-related genes was shown in Figure 1D. Additionally, KEGG enrichment analysis revealed the enrichment of several malignancy-associated signaling pathways (Figure 1E).

The PPI networks of 186 EMT-related DEGs were reconstructed using the STRING database. The resulting network of interaction relationships consisted of 161 nodes and 917 edges (with a minimum required interaction score >0.4), indicating the complexity of EMT in PCa progression (Figure 1F). The most important module was identified using the Cytotype parameter Settings with the MCODE plug-in (Figure 1G). According to the KEGG enrichment analysis, the genes in this module were found to be highly correlated with PCa (Figure 1H). This implies that these genes may potentially contribute significantly to the progression of PCa.

### 3.2 Consensus clustering of PCa patients based on the expression of EMT-related DEGs and identification of EMT regulator pattern-related gene clusters

**FIGURE 2:**
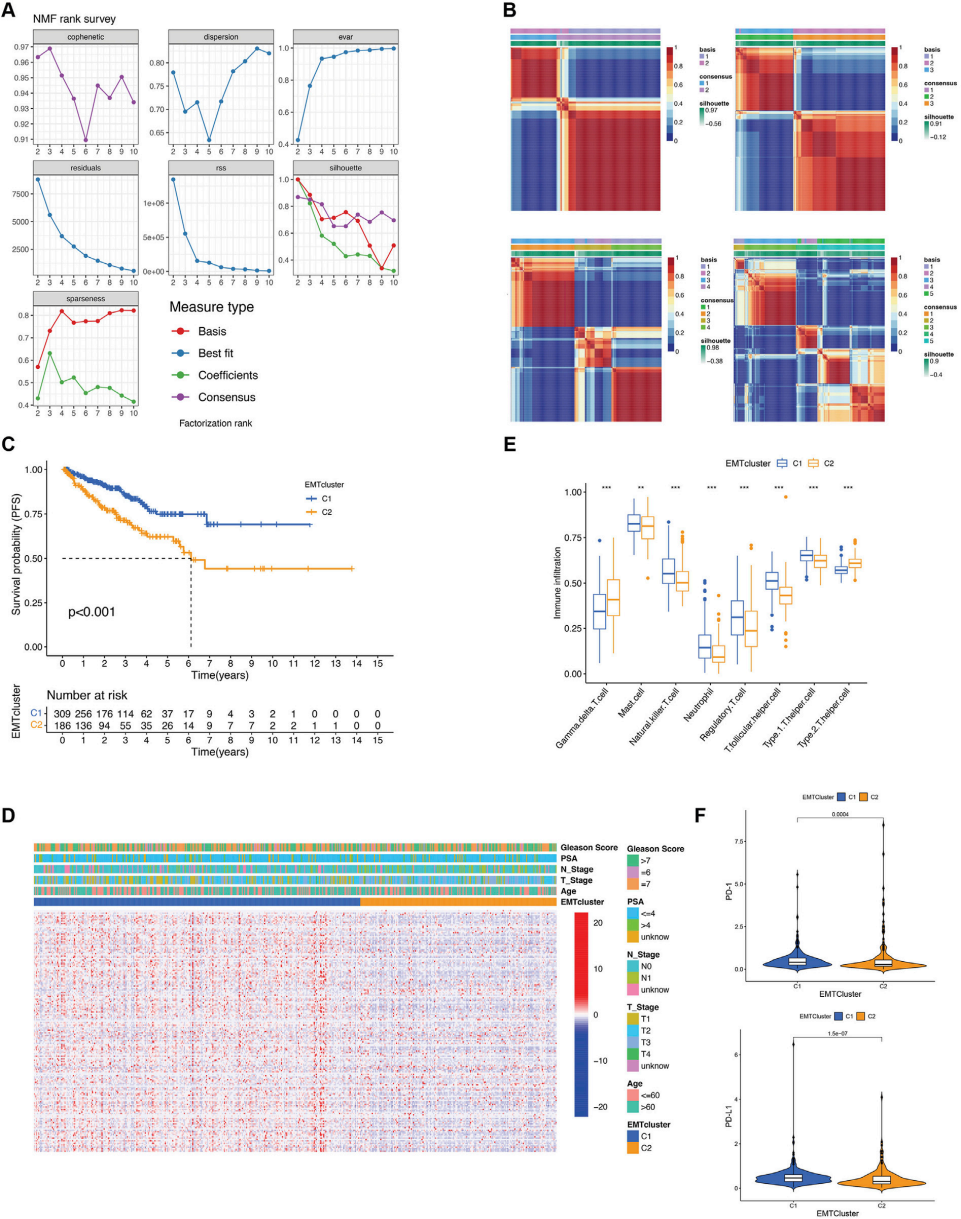
Identification of distinct EMT regulation patterns using the NMF algorithm. **(A)** The NMF rank survey with the different number of clusters (rank k = 2–10). **(B)** The EMT regulation patterns with the different number of clusters (rank k = 2–4), indicating k = 2 was identified as the optimal value. **(C)** Kaplan-Meier survival analysis was applied to analyze the PFS prognosis of PCa patients between the two EMT regulation patterns. **(D)** The distributions in clinicopathologic characteristics between the two EMT regulation patterns. **(E)** Boxplot of relative infiltrating immune cell abundance between the two EMT regulation patterns. **(F)** Difference of PD1 and PD-L1 between the two EMT regulation patterns.

Consensus clustering was conducted using the NMF algorithm to differentiate PCa patients with different EMT regulation patterns based on the expression of 186 EMT-related DEGs. The NMF rank survey, including cophenetic and dispersion (Figure 2A), and the consensus matrix heatmap (k = 2–5, Figure 2B), showed that k = 2 was the optimal parameter to categorize the PCa patients into two distinct regulation patterns. There were 309 cases in EMT cluster 1 and 186 cases in EMT cluster 2. Characteristics of cluster 1 and cluster 2 from TCGA data were listed in Table 1. Prognostic analysis revealed that EMT cluster 1 had a more significant survival advantage than EMT cluster 2 (Figure 2C). The heatmap of the expression of EMT-related DEGs demonstrated the distinction between the two clusters (Figure 2D). Next, we utilized the ssGSEA method to analyze the differences in immune cell composition between the two EMT clusters. The study results indicate that the abundance of immune cells, including mast cells, natural killer T cells, neutrophils, regulatory T cells, and follicular/Type1 helper T cells, was significantly higher in EMT cluster 1. On the other hand, the abundance of Gamma-delta (γδ) T cells and Type2 helper T cells was significantly higher in EMT cluster 2 (Figure 2E). Additionally, tumors in EMT cluster 1 showed increased expression of PD-1 and PD-L1 (Figure 2F). These results indicate that two EMT regulatory patterns are distinct in terms of clinical features and immune infiltration levels, suggesting that PCa cases can be classified according to EMT-related genes.

To demonstrate the heterogeneity of different EMT regulator patterns, we compared gene expression profiles between the two clusters and identified 2,512 genes associated with the EMT regulator pattern (Supplementary Figure S1A). Subsequently, a univariate Cox regression was conducted on these genes and identified 873 genes significantly associated with PFS (Supplementary Figure S1B). Consistent with the clustering of EMT regulation patterns, NMF cluster analysis based on the expression of these 873 genes explicitly categorized PCa patients into four clusters, which we termed EMT gene clusters 1-4, respectively (Supplementary Figures 1C, D **)**. Of the 495 patients with PCa, 84 were clustered into gene cluster 1, which was linked to the best prognosis among the four gene clusters, while patients in gene cluster 2 (n = 178) experienced the worst outcome (Supplementary Figure S1E). The expression profiles of the 873 genes that regulate EMT patterns and their clinical characteristics were illustrated in a heatmap (Supplementary Figure S1F). Significant differences in the expression of EMT-related DEGs were observed in the most important module of the four EMT gene clusters, which is in accordance with the predicted findings of the EMT patterns (Supplementary Figure S1G).

### 3.3 Construction and validation of the EMT prognostic signature

**FIGURE 3:**
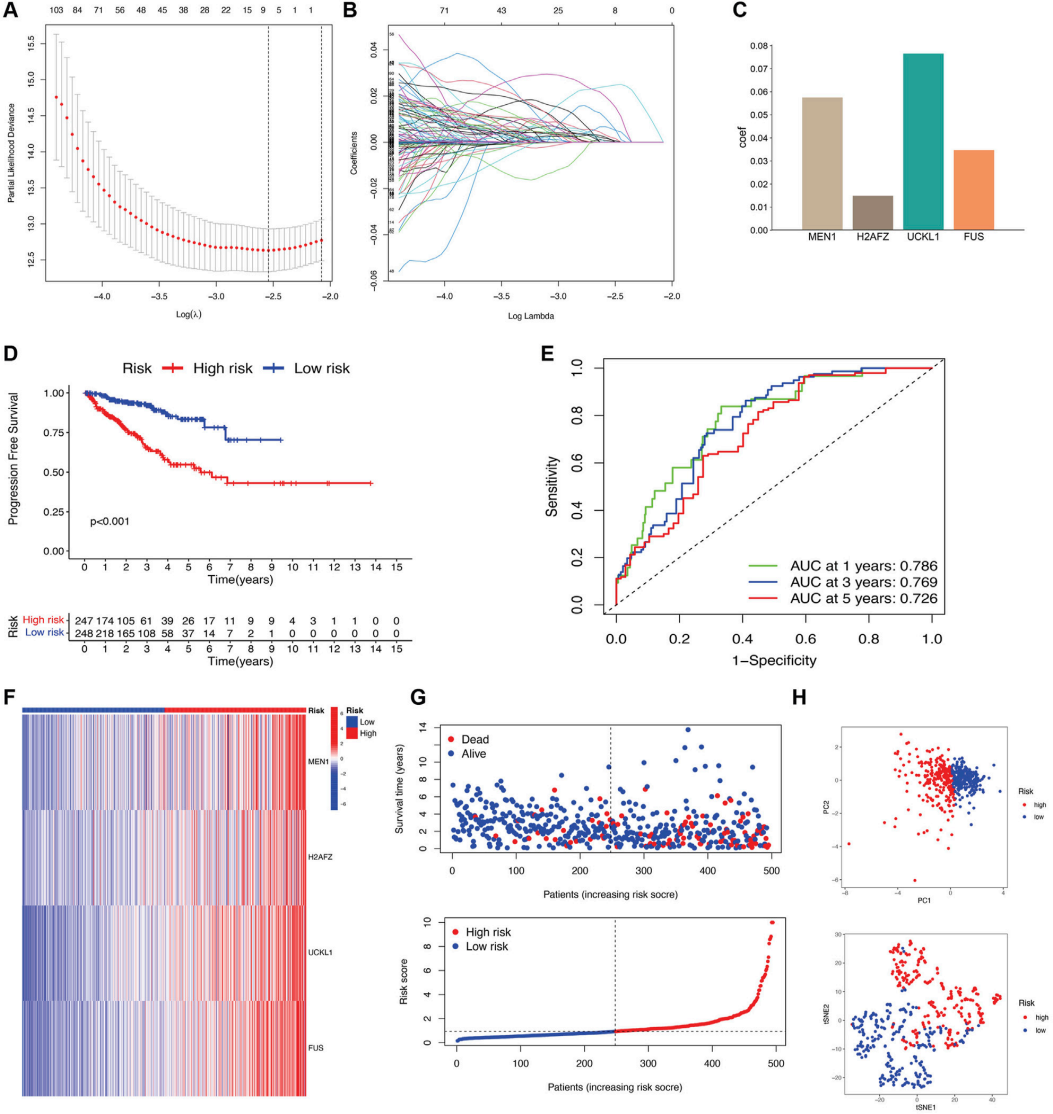
Construction of a novel EMT prognostic signature in the TCGA cohort. **(A)** LASSO regression analysis identified candidate genes with a minimum lambda value. **(B)** Coefficients of the LASSO analysis. **(C)** Coefficients of the final four genes for the construction of the EMT prognostic signature in the Multivariate Cox regression analysis. **(D)** Kaplan-Meier survival analysis showing a significant PFS difference between low- and high-risk groups. **(E)** Time-dependent ROC curves analysis. **(F)** Heatmap of four key prognostic gene expressions of low- and high-risk groups. **(G)** Distribution of risk scores and patient survival of low- and high-risk groups. **(H)** PCA and t-SNE plot displaying the distribution of low- and high-risk groups.

To quantify individual patients’ EMT scores, we constructed an EPS considering the heterogeneity and complexity of PCa patients. We included 873 EMT regulator pattern-related prognostic genes in the LASSO-Cox regression model and screened out nine genes based on the minimum value of λ (Figures 3A,B). A multivariate Cox regression analysis was conducted on nine genes using the Akaike information criterion value. Four genes (*MEN1*, *H2AFZ*, *UCKL1*, and *FUS*) were selected to establish an EPS (Figure 3C). The risk score was derived with the expression levels and coefficients of the above four genes: risk score = (0.0575 * *MEN1*) + (0.0150 *H2AFZ) + (0.0765 * *UCKL1*) + (0.0347 * *FUS*).

Next, the patients were classified into low-risk and high-risk groups according to their median risk scores. According to the Kaplan-Meier curve, PCa patients in the low-risk group had better PFS (*P* < 0.001) (Figure 3D). The area under the ROC curve (AUC) values for 1-, 3-, and 5-year survival were 0.786, 0.769, and 0.726, respectively (Figure 3E). The gene expression profiling heatmap showed a significant increase in the expression of four genes in the high-risk stratification (Figure 3F). The plot of survival status and risk score revealed that the high-risk group had a worse PFS rate (Figure 3G). Additionally, we demonstrated the bidirectional distribution of PCa patients in different groups by PCA and t-SNE analyses (Figure 3H).

To verify the prognostic potential of the EPS, a similar analysis was performed on the GSE116918 dataset. Patients were categorized into two groups based on the formula used in the TCGA cohort. The high-risk group exhibited lower survival rates (*P* < 0.001) (Supplementary Figure S2A). The AUC values of 0.992, 0.873, and 0.613 predicted 1-, 3-, and 5-year BCR, respectively (Supplementary Figure S2B). Additionally, our results were consistent with those of the TCGA dataset in terms of the distribution plots of the expression of the 4 genes, risk score and survival status, PCA, and t-SNE analyses (Supplementary Figures 2C–E). The results indicate that the EPS is a significant biomarker for predicting the survival of PFS and BCR, with potential clinical applications.

### 3.4 Clinical evaluation of the EMT prognostic signature

To investigate the correlation between the risk scoring system and clinicopathological characteristics, we analyzed differences in various stratified features. The findings revealed that in the TCGA cohort, patients with older age (*P* < 0.05), higher T-stage (*P* < 0.01), and higher N-stage (*P* < 0.001) had higher risk scores (Supplementary Figures 3A, B). Furthermore, patients in gene cluster 2 and cluster 1 had the highest and lowest risk score, which was consistent with their survival outcomes. Next, we investigated the predictive value of risk scores for various clinical characteristics. Furthermore, stratified prognostic analysis revealed that the high-risk group had a worse prognosis in all subgroups except for the N1 stage (Supplementary Figure S3C).

**FIGURE 4:**
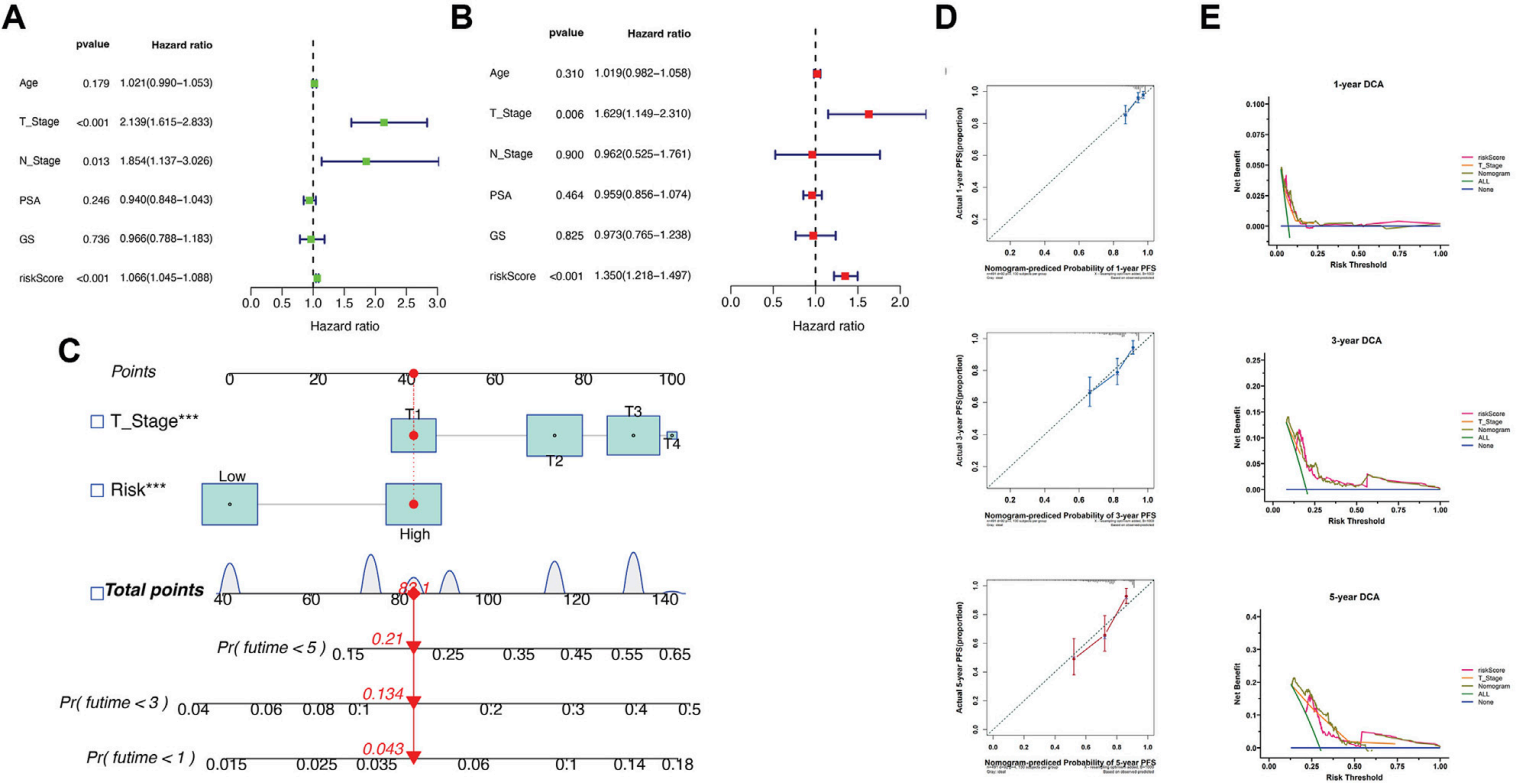
Development of a comprehensive nomogram predicting the PFS of PCa patients. **(A,B)** Univariate and multivariate Cox analyses of the EMT prognostic signature and clinicopathological features. **(C)** A nomogram model was developed to predict patients’ prognosis quantitatively. **(D)** The calibration curves showing good agreement between the anticipated and actual probability of 1-, 3-, and 5-year survival rates. **(E)** Decision curve analysis of the nomogram for 1-, 3-, and 5-year survival.

Univariate and multivariate Cox regression analyses were conducted to determine the predictiveness of risk scores. The study found that the T-stage and risk score are independent prognostic indicators of PFS in PCa patients (Figures 4A,B). A nomogram using these independent indicators (T-stage and risk score) was developed to predict PFS (Figure 4C). The calibration curves showed good agreement between the predicted and actual observations of 1-, 3-, and 5-year PFS (Figure 4D). Additionally, the DCA demonstrated that the nomogram provided a greater net benefit compared to other independent factors, indicating its reliability (Figure 4E). To summarize, the predictive nomogram can accurately predict an individual’s survival risk and aid in clinical management.

### 3.5 Mutation status and genomic heterogeneity associated with the EMT prognostic signature

Since genetic mutations and genomic heterogeneity are the conditions for oncogenesis, we visualized somatic mutations of PCa samples based on the EPS, which showed that *SPOP* (11%), *TP53* (11%), and *TTN* (10%) had the highest mutation frequencies (Supplementary Figure S4A). Analysis of variance showed that TMB was significantly higher in the high-risk group than in the low-risk group (Supplementary Figure S4B). Spearman’s correlation analysis revealed a positive correlation between TMB and the risk score (Supplementary Figure S4C). The Kaplan-Meier curve also demonstrated that the low-TMB group had a better prognosis (*P* = 0.01) (Supplementary Figure S4D). By combining TMB and the risk score, PCa patients were categorized into four subgroups for survival assessment. The survival analysis showed that patients with low TMB and low risk had the best prognosis (*P* < 0.01) (Supplementary Figure S4E), which validates the EPS. Correlation analysis subsequently revealed that the risk score was positively correlated with HRD (R = 0.37, *P* = 3.5e-16), LOH (R = 0.34, *P* = 3.3e-14), MSI (R = 0.28, *P* = 1.7e-10), and purity (R = 0.42, *P* = 1.3e-07), but not with MATH, neoantigen, and polity (Supplementary Figure S4F). These results may reflect the practical application of the EPS in exploring PCa heterogeneity.

### 3.6 Immune landscape and therapeutic responses associated with the EMT prognostic signature

**FIGURE 5:**
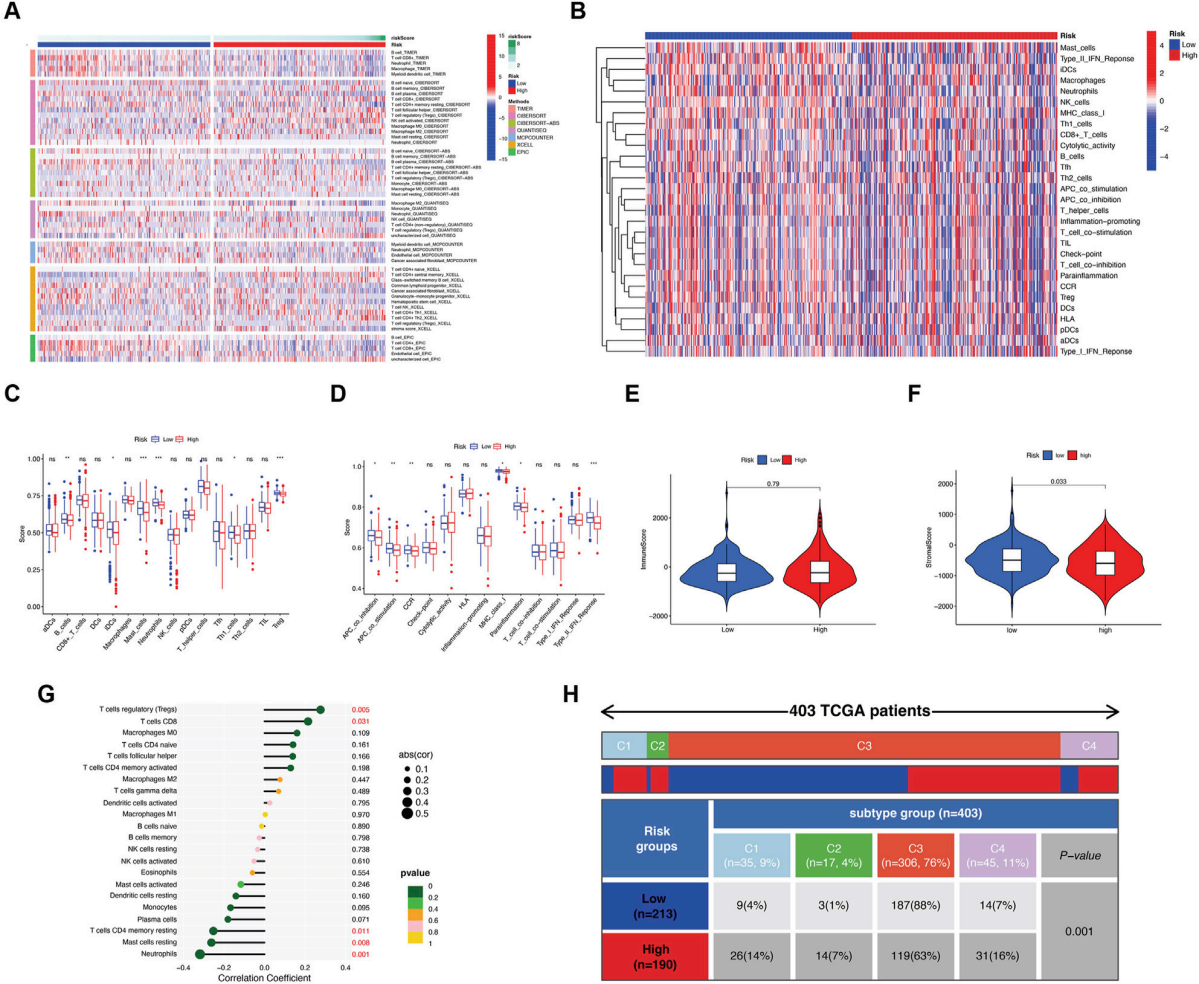
Potential effects of EMT prognostic signatures on tumor immune microenvironment. **(A)** Heatmap showing the abundance of the tumor-infiltrating immune cells using seven algorithms between the two risk groups. **(B)** Heatmap of the relationship of different groups and 29 immune signatures identified by a previous study. **(C)** The 16 immune cell infiltration levels between the two risk groups. **(D)** The 13 immune function levels in two risk groups. **(E,F)** Comparison of the immune scores and stromal scores between the two risk groups. **(G)** Correlation between risk score and 22 tumor-infiltrating immune cells obtained using the CIBERSORT method. **(H)** The distribution of two risk groups from PCa in the pan-cancer immune subtypes.

Various levels of immune cell infiltration play a crucial role in the development and progression of cancer. We conducted an analysis of immune cell infiltration using seven algorithms to investigate the correlation between the risk score and the tumor immune microenvironment in patients with PCa (Figure 5A). Besides, the study assessed the abundance of tumor-infiltrating immune cells and immune functions with the ssGSEA algorithm (Figure 5B). Moreover, the results suggest that there may be a difference in immune cell infiltration that leads to altered immune function. The results showed that the low-risk group had significantly higher immune cell and function scores than the high-risk group (Figures 5C,D). Although the stroma score was higher in the low-risk group, there was no significant difference in immune scores between the two subgroups (Figures 5E,F). To further clarify the effect of the risk score on 22 tumor-infiltrating immune cells (TIICs), we calculated the TIIC levels for each sample using the CIBERSORT method. The risk score was shown to be positively associated with Tregs and CD8 T cells, but negatively associated with resting memory CD4 T cells, resting mast cells, and neutrophils (Figure 5G). Upon comparing the relationship between the risk score and the defined immune categories, as per the study conducted by Thorsson et al. (Thorsson et al., 2018), we observed a distribution difference in the proportions of C3 (inflammatory) and C4 (lymphocyte depleted) between the low-risk and high-risk groups (*P* = 0.001) (Figure 5H). Specifically, C3 was prevalent in the low-risk group, which was significantly associated with good survival, consistent with our findings. C4 was highly clustered in the high-risk group. This result not only reveals the unique features of the PCa immune microenvironment but also adds to previous studies.

**FIGURE 6:**
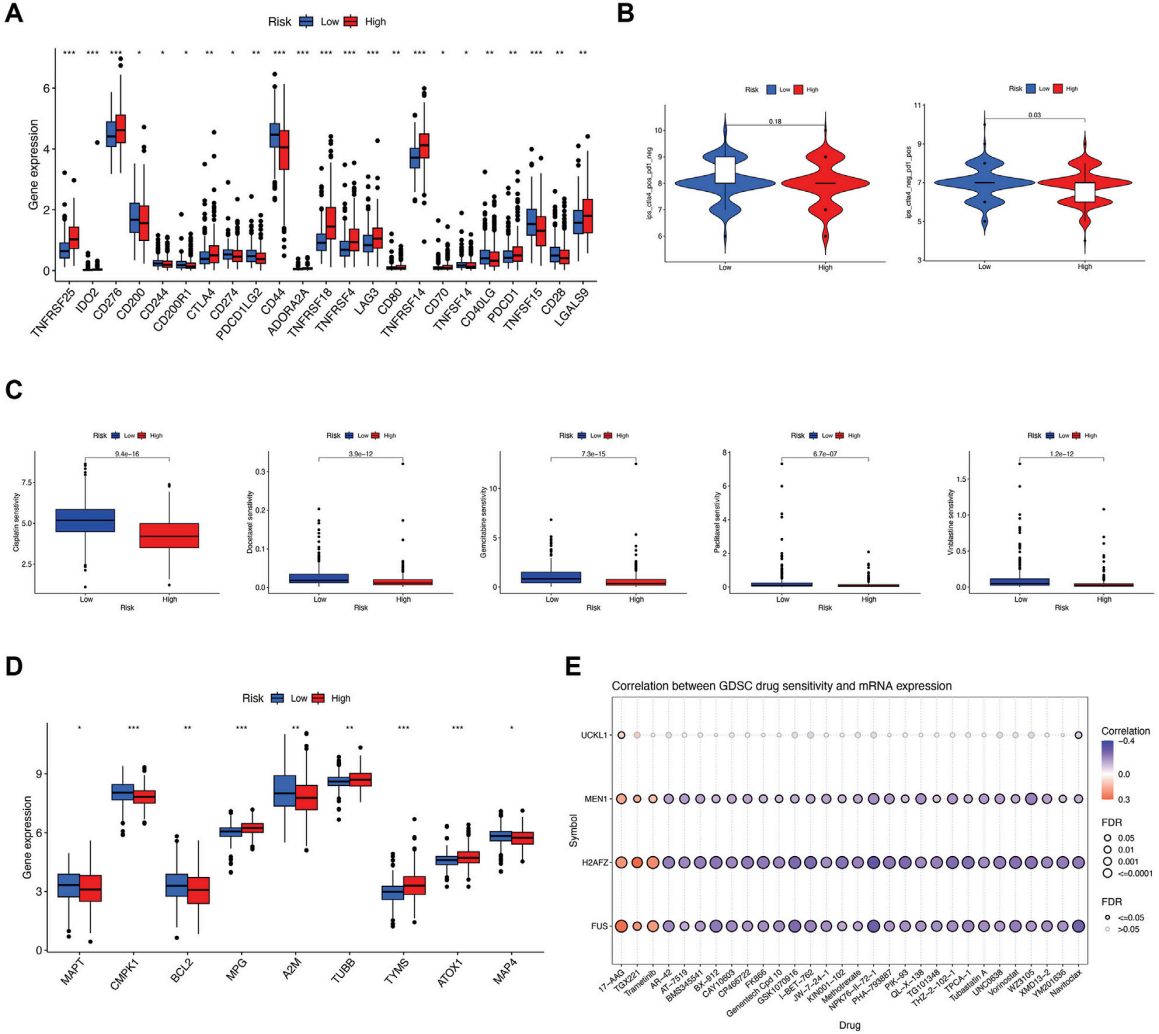
Potential effects of EMT prognostic signatures on immunotherapy and chemotherapy. **(A)** Difference in the expression of immune checkpoint inhibitors between the two risk groups. **(B)** Difference in the immunophenoscore (IPS) between the low- and high-risk groups stratified by both CTLA4 and PD-1. **(C)** Drug sensitivity of Cisplatin, Docetaxel, Gemcitabine, Paclitaxel, and Vinblastine between the two risk groups. **(D)** Difference in expression of target genes in anticancer drugs between the two risk groups. **(E)** The correlation of the four key prognostic genes with the resistance to common chemotherapeutics using the GDSC database. Red indicates a positive correlation, which means that the high expression of the gene is resistant to the drug. The opposite is true for a negative correlation.

Considering the significance of tumor immunotherapy based on immune checkpoint inhibitors (ICIs), we further investigated the differential expression of immune checkpoints between the two groups. In the high-risk group, 13 out of 46 immune checkpoints, including PD-1 (PDCD1) and CTLA4, were upregulated, while 10 immune checkpoints, including PD-L1 (CD274) and PD-L2 (PDCD1LG2), were downregulated (Figure 6A). Additionally, we investigated the immune-related progression-free survival of patients undergoing various treatments. Our findings indicate that in the CTLA4-negative/PD-1-positive subgroups, patients with a low-risk score had a higher IPS (*P* = 0.03). This higher IPS predicted a more favorable therapeutic outcome (Figure 6B).

Furthermore, to determine the feasibility of using the risk score to personalize chemotherapy for PCa, we explored whether risk scores correlated with IC50 values for five commonly used medications. As shown in Figure 6C, Cisplatin, Docetaxel, Gemcitabine, Paclitaxel, and Vinblastine exhibited greater sensitivity in the low-risk group. From the gene expression data obtained from the DrugBank database, it was observed that nine genes targeted by these drugs had different expression patterns between the two groups (Figure 6D). We next conducted the correlation analysis between risk prognostic-related four key genes and the clinical efficacy of PCa treatments. By analyzing the drug response data from the GDSC database, we noticed that these four genes were significantly linked with sensitivity or resistance to multiple therapeutic drugs and molecular inhibitors using Spearman’s correlation coefficient. The results indicated that the high expression of *MEN1*, *H2AFZ, UCKL1,* and *FUS* produced resistance to 17-AAG (a selective HSP90 inhibitor), and *MEN1, H2AFZ,* and *FUS* also produced resistance to TGX221 (a selective PI3K inhibitor) and Trametinib (a selective MEK inhibitor). Simultaneously, *MEN1*, *H2AFZ UCKL1,* and *FUS* were all sensitive to Navitoclax (ABT-263), an effective Bcl-2 inhibitor. *MEN1*, *H2AFZ,* and *FUS* were also sensitive to Tubastatin (a selective HDAC6 inhibitor), Vorinostat (an HDAC inhibitor), and other molecular inhibitors as well as drugs (Figure 6E).

### 3.7 Verification of the protein and mRNA expression levels of four key prognostic genes

**FIGURE 7:**
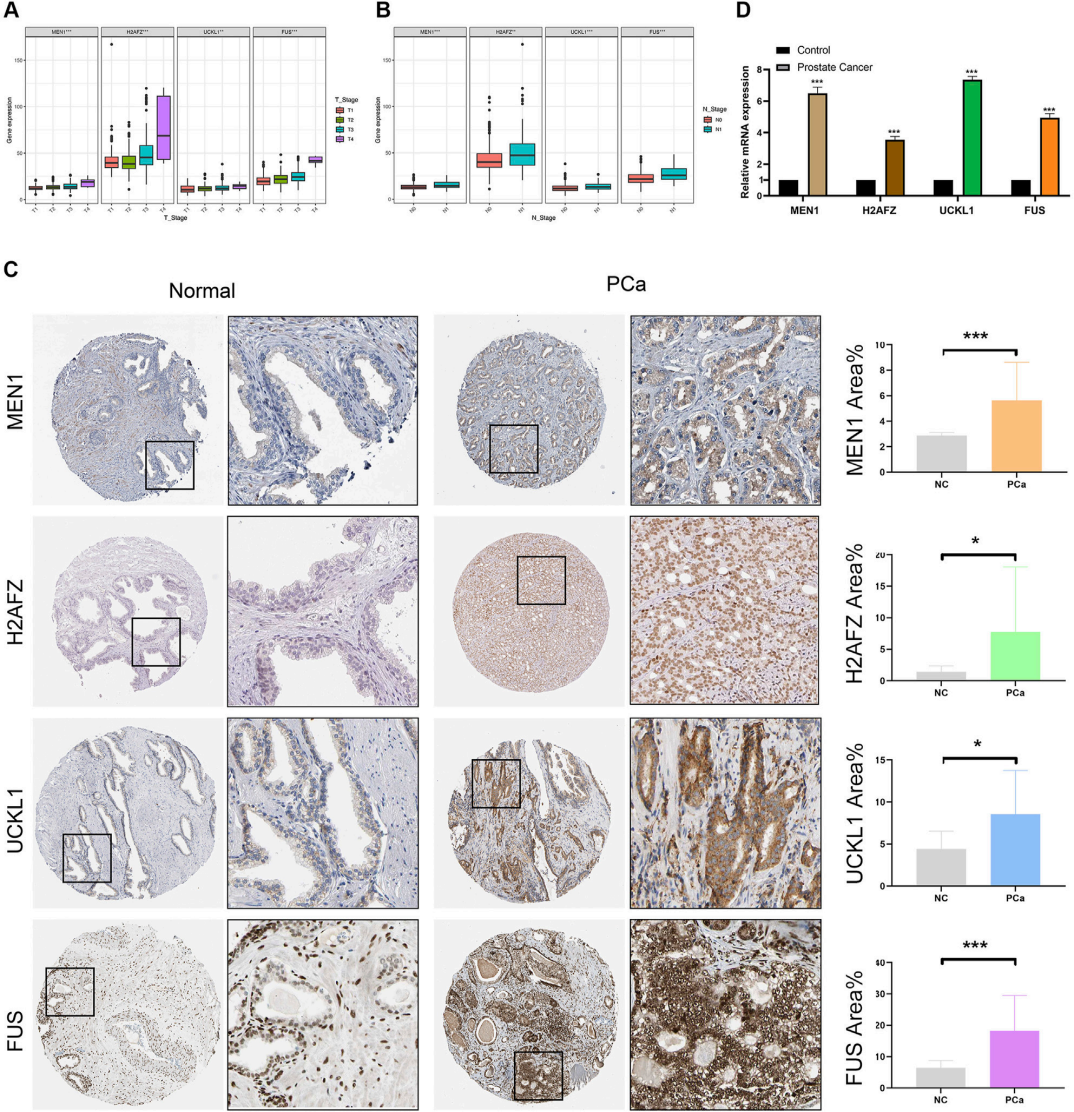
Validation of protein and mRNA expression of four key prognostic genes (*MEN1*, *H2AFZ*, *UCKL1*, and *FUS*) that comprised the EMT prognostic signature. **(A,B)** The expression levels of four key prognostic genes in different T stage and N stage. **(C)** Assessment of protein levels of four key prognostic genes using data from the Human Protein Atlas. Histograms represent MEN1, H2AFZ, UCKL1, and FUS area% expression in PCa and NC groups. IHC results showed that the protein levels of these genes are higher in PCa tissues compared to normal prostate tissues. **(D)** qRT-PCR results showed that four key prognostic genes were highly expressed in PCa tissues compared with the adjacent normal prostate tissues.

As previously mentioned, the EPS was based on four key prognostic genes, including *MEN1*, *H2AFZ*, *UCKL1*, and *FUS*. It is noteworthy that *MEN1* and *H2AFZ* are EMT-related genes. We associated the expression levels of these four key genes with clinicopathological features and found that their expression was inversely correlated with T- and N-stage (Figures 7A,B). IHC were introduced to examine the protein expression levels of the four genes in the HPA database, the results revealed a significant increase in their protein expression in PCa tissues compared to normal prostate tissues (all *P* < 0.05) (Figure 7C). Furthermore, we conducted a real-world experiment to detect the relative mRNA expression levels using qRT-PCR. The study confirmed that the expression levels of *MEN1*, *H2AFZ*, *UCKL1*, and *FUS* were higher in PCa than in adjacent normal prostate tissues (*P* < 0.05) (Figure 7D). This finding is consistent with the bioinformatic analyses.

## 4 Discussion

Compared to early-stage PCa, advanced or metastatic PCa does not respond well to various treatments, and is often associated with considerable poor prognosis and shortened overall survival (OS). The limitations of traditional treatments for advanced or metastatic PCa, such as androgen deprivation therapy (ADT) and chemotherapy, remind us of the need to investigate novel treatments that can achieve durable disease control and long-term survival benefits. Recent studies suggest that the initiation, progression, metastasis, and treatment resistance of PCa involve the interaction between tumor cells and the host immune system induced by EMT. Therefore, immunotherapy should be considered as a potential treatment option for PCa management. However, unlike other urologic malignancies such as bladder cancer (BCa) or kidney cancer, the response to immunotherapy in PCa is insufficient. Investigating novel prognostic signatures of EMT-related genes is critical for predicting survival and treatment responses of PCa immunotherapy.

In this study, patients were classified according to the expression of 186 EMT-related DEGs. Of 2,512 differentially expressed EMT regulation pattern-related genes, 873 were identified as significantly associated with PFS and had distinct EMT regulation patterns between the two clusters. A second clustering analysis was conducted using the NMF algorithm to divide the patients into four distinct clusters (C1, C2, C3, and C4) based on the expressions of 873 differentially expressed genes related to EMT regulation patterns. From these 873 genes, four were identified to construct an EPS through LASSO and Cox regression analysis. The EPS divided PCa patients into low- and high-risk groups and demonstrated outstanding survival prediction efficacy for patients with PCa. Its prognostic utility was confirmed in an independent validation cohort (GSE116918). ROC analysis evaluated the time-associated outcomes of the EPS in PCa patients from the TCGA cohort. The analysis showed that the prediction efficacy in short-term survival was relatively better than in long-term survival. Similar results were observed in the external validation cohort (GSE116918), confirming the prognostic utility of the EPS in patients with PCa. The AUC value obtained from the EPS may not have reached its maximum potential due to the complex mechanisms involved in tumor progression and metastasis in PCa, which are influenced by various factors beyond the EMT-related genes. Therefore, other important genes may also contribute to this pathological process. Besides, our study found that EPS was an independent risk factor for worse outcomes, in addition to T-stage and N-stage. However, M-stage and Gleason scores were not found to be independent prognostic factors for patients with PCa. This may be due to EMT being more associated with tumor occurrence and lymph node metastasis than with PCa metastasis and Gleason score. Therefore, EPS constructed using EMT-related genes, T-stage, and N-stage can serve as an independent predictor. M-stage and Gleason score were not included.

The EPS comprises four genes: *Menin 1* (*MEN1*), *Histone H2A Z* (*H2AFZ*), *Uridine-cytidine kinase 1 like 1* (*UCKL1*), and *FUS RNA binding protein* (*FUS*), which have been reported to be associated with several malignancies. *MEN1* has been reported to act as an oncogenic factor in various solid tumors, such as hepatocellular carcinoma (Kempinska et al., 2018), breast cancer (Dreijerink et al., 2017), and PCa (Kim et al., 2022). Kim T. et al. discovered that menin is involved in tumor cell growth and metastasis in PCa cells with low or deficient levels of androgen receptor (AR) (Kim et al., 2022). Cherif C. et al. found that menin is overexpressed in high-grade PCa and castration-resistant prostate cancer (CRPC). Besides, elevated *MEN1* mRNA expression is linked to shorter BCR-free survival and OS. Inhibiting menin could suppress CRPC cell proliferation and restore chemosensitivity (Cherif et al., 2022). *H2AFZ* and *H2AFV* are two non-allelic genes responsible for encoding two distinct isoforms of H2A.Z, which is one of the histone H2A variants in mammalian cells with 60% similarity with canonical histone H2A (Sevilla and Binda, 2014). Studies have shown that overexpression of H2A.Z promotes proliferation in breast cancer, BCa, and PCa (Vardabasso et al., 2014). Ito S. et al. discovered that the MRG domain binding protein enhances the expression of specific AR target genes, such as kallikrein-related peptidase 3 (also known as prostate-specific antigen) and TMPRSS2, by activating AR-associated enhancer and promoter regions through acetylation of histone variant H2A.Z at the AR binding site. This may explain H2AFZ’s oncogenic role in PCa disease (Ito et al., 2018). S Many reports have revealed the oncogenic effects of *UCKL1* in hepatocellular carcinoma (Yu et al., 2019), breast cancer (Kovalevska et al., 2021), and colorectal cancer (Wu et al., 2023). However, there are few studies on the role of *UCKL1* in PCa. Kovalevska L. et al. have found that *UCKL1* was overexpressed in both blood sera and tumor tissue of PCa patients (Kovalevska et al., 2022). Similarly, Cheng W. et al. investigated PCa-related genes and identified *UCKL1* as one of the most important differentially expressed genes in PCa disease (Cheng et al., 2014). In contrast, the role of FUS in PCa has been extensively studied. Experiments conducted by Feng Y. et al. confirmed that FUS promotes the proliferation and migration of PCa cells (Feng et al., 2019). However, other researchers have found that FUS has an antitumor effect in PCa disease (Wang et al., 2020; Abd Raboh et al., 2021). This study clarifies the prognostic value of four EMT-related genes in PCa. Further investigation is needed to understand the mechanism of these four genes in PCa.

TME comprises cancer cells, stromal cells, immune cells, extracellular matrix, and associated acellular components. Infiltrating immune cells are vital in tumorigenesis, metastasis, and regulation of anti-cancer immunity, making them a potential therapeutic target (Pitt et al., 2016). Our study analyzed TMB levels in different EPS-based risk score groups. We found that patients in the low-risk group had lower TMB levels and better prognosis than those in the high-risk group. High TMB is generally associated with a better response to ICIs, such as anti-PD-1 therapy. This association is supported by pooled analyses of 27 tumor types (Yarchoan et al., 2017). However, the present study’s seemingly contradictory finding may reflect the fact that the association of TMB with survival outside of the immunotherapy context is poorly understood. Additionally, the broad applicability of high TMB as a biomarker of response across all solid malignancies is unclear. This research aims to investigate the ideal biomarkers for guiding the selection and management of immunotherapy. Under the selective pressure of immunotherapy, persisting mutations are preserved in tumor development. Tumors with elevated TMB exhibit a more inflammatory TME (Niknafs et al., 2023). Our study showed a high EPS-based risk score was positively associated with T cells and macrophages, while negatively associated with mast cells and neutrophils. This finding is consistent with previous reports, as several studies have examined the function of immune cells that infiltrate tumors. Four studies have shown that increased infiltration of M1 macrophages is associated with poor disease outcomes in PCa (Wu et al., 2020; Zhang et al., 2020). One study investigated the role of M0 macrophages in PCa prognosis and reported increased infiltration of M0 macrophages into the prostate tissue of patients with a high risk of cancer-specific death, disease-free survival, or biochemical recurrence (Zhang et al., 2020). Other studies have shown that increased infiltration of mast cells into cancerous tissue is associated with improved prognosis or reduced risk score for PCa (Zhang et al., 2021; Zhao et al., 2021). However, conflicting results have been observed regarding the effects of T cells and neutrophils on PCa, as some studies have found that these immune cells can either promote or suppress cancer (Hu et al., 2015; Masucci et al., 2019; Fu et al., 2021; Zhao et al., 2021).

Despite the above-mentioned strengths of our study, several limitations existed in the present study. Firstly, our study was retrospective and relied on information from a public database for both modeling and validation. Therefore, future prospective studies are warranted to evaluate the clinical utility of our model in patients with PCa. This study only explored the relative expression levels of the four target genes in PCa specimens. Comprehensive functional experiments should be conducted subsequently to elucidate the detailed mechanisms of the four target genes.

## Data Availability

The datasets used and/or analyzed during the current study are available from the corresponding authors upon reasonable request. The database links for the data used in this study are as follows: The Cancer Genome Atlas database (TCGA, https://portal.gdc.cancer.gov/), the Gene Expression Omnibus database (GEO, https://www.ncbi.nlm.nih.gov/geo/), dbEMT 2.0 database (https://dbemt.bioinfo-minzhao.org/index.html), STRING database (http://string-db.org), the Cancer Immunome Atlas database (TCIA, https://tcia.at/home), the DrugBank database (https://go.drugbank.com/), the Gene Set Cancer Analysis database (GSCA, https://guolab.wchscu.cn/GSCA), and the Human Protein Atlas database (HPA, https://www.proteinatlas.org/).
