# Supplementary material for: Identification of epithelial-mesenchymal transition prognostic signature associated with prognosis, tumor microenvironment, and therapeutic effect in prostate cancer
Source: Front Genet. 2025 Aug 11;16:1539745. doi: 10.3389/fgene.2025.1539745 (PMC12375637; doi:10.3389/fgene.2025.1539745)
Supplement: Supplementary file 1 [file Table1.docx]

Supplementary Material

## Supplementary Figures Legend

**Supplementary Figure 1. Identification of distinct EMT-related gene clusters using the NMF algorithm.** (A) Volcano map of 2512 differentially expressed EMT regulator pattern-related genes between the two EMT regulation patterns. (B) 873 of 2512 genes were significantly associated with PFS in the univariate Cox regression. (C) The NMF rank survey with the different number of clusters (rank k = 2 to 10). (D) The EMT gene clusters with a different number of clusters (rank k = 2 to 4), indicating k = 4 was identified as the optimal value. (E) Kaplan-Meier survival analysis was applied to analyze the PFS prognosis of PCa patients between the four EMT gene clusters. (F) The distributions in clinicopathologic characteristics between the four EMT gene clusters. (G) Difference of EMT-related gene expression across four gene clusters.

**Supplementary Figure 2. Construction of a novel EMT prognostic signature in the** **GSE116918 cohort.** (A) Kaplan-Meier survival analysis showing a significant BCR difference between low- and high-risk groups. (B) Time-dependent ROC curves analysis. (C) Heatmap of four key prognostic gene expressions of low- and high-risk groups. (D) Distribution of risk scores and patient survival of low- and high-risk groups. (E) PCA and t-SNE plot displaying the distribution of low- and high-risk groups.

**Supplementary Figure 3. Clinical correlation and stratified survival analysis of EMT prognostic signature in PCa patients.** (A) The landscape of EMT prognostic signature and clinicopathological features (Age, T stage, N stage, PSA, and Gleason Score). (B) The distribution and the difference of age, T stage, and N stage in two groups. (C) Kaplan-Meier survival analysis of patients in two groups using EMT prognostic signature stratified by different clinicopathological features.

**Supplementary Figure 4. Mutation and genomic heterogeneity based on the EMT prognostic signature in PCa patients.** (A) Oncoplot of top 30 mutation genes in PCa patients. (B) Boxplot revealing the difference in TMB between the two groups. (C) Spearman correlation analysis between risk score and TMB. (D) Kaplan-Meier survival analysis of patients in low- and high TMB groups. (E) Kaplan-Meier survival analysis of distinct groups stratified by both TMB and risk score. (F) Correlation between risk score and different genomic heterogeneities.
